# Drug target prediction using adverse event report systems: a pharmacogenomic approach

**DOI:** 10.1093/bioinformatics/bts413

**Published:** 2012-09-03

**Authors:** Masataka Takarabe, Masaaki Kotera, Yosuke Nishimura, Susumu Goto, Yoshihiro Yamanishi

**Affiliations:** ^1^ Bioinformatics Center, Institute for Chemical Research, Kyoto University, Gokasho, Uji, Kyoto 611-0011, Japan and; ^2^Division of System Cohort, Medical Institute of Bioregulation, Kyushu University, 3-1-1 Maidashi, Higashi-ku, Fukuoka, Fukuoka 812-8582, Japan

## Abstract

**Motivation:** Unexpected drug activities derived from off-targets are usually undesired and harmful; however, they can occasionally be beneficial for different therapeutic indications. There are many uncharacterized drugs whose target proteins (including the primary target and off-targets) remain unknown. The identification of all potential drug targets has become an important issue in drug repositioning to reuse known drugs for new therapeutic indications.

**Results:** We defined pharmacological similarity for all possible drugs using the US Food and Drug Administration's (FDA's) adverse event reporting system (AERS) and developed a new method to predict unknown drug–target interactions on a large scale from the integration of pharmacological similarity of drugs and genomic sequence similarity of target proteins in the framework of a pharmacogenomic approach. The proposed method was applicable to a large number of drugs and it was useful especially for predicting unknown drug–target interactions that could not be expected from drug chemical structures. We made a comprehensive prediction for potential off-targets of 1874 drugs with known targets and potential target profiles of 2519 drugs without known targets, which suggests many potential drug–target interactions that were not predicted by previous chemogenomic or pharmacogenomic approaches.

**Availability:** Softwares are available upon request.

**Contact:** yamanishi@bioreg.kyushu-u.ac.jp

**Supplementary Information:** Datasets and all results are available at http://cbio.ensmp.fr/~yyamanishi/aers/.

## 1 INTRODUCTION

Most drugs are molecules that interact and interfere with an appropriate protein target implicated in a disease of interest. Drugs may also interact with additional proteins (off-targets hereafter) that are not their primary therapeutic targets, resulting in unexpected side effects. Drug side effects are complex phenomena attributed to many molecular scenarios (e.g. metabolism disorder, downstream pathway perturbations), among which the interaction with off-target proteins is the most important cause (Blagg, 2006; Whitebread *et al.*, 2005). Unexpected drug activities derived from off-targets are usually undesired and harmful; however, they can occasionally be beneficial and lead to different therapeutic indications. For example, sildenafil (Viagra) was developed to treat angina, but it is now used for the treatment of erectile dysfunction.

There are many drugs whose target proteins (including the primary target and off-targets) have not yet been characterized. The identification of all potential targets for a given drug has become an important issue in drug repositioning to reuse known drugs for new therapeutic indications. Experimental testing to identify drug–target interactions is a very expensive and time-consuming process, and thus there is a strong incentive to develop new *in silico* prediction methods, which will enable to limit experimental testing.

In recent years, the field of chemogenomics has rapidly gained importance, primarily exploring the relationship between the chemical space of possible compounds and the genomic space of possible proteins (Dobson, 2004; Kanehisa *et al.*, 2006; Stockwell, 2000). A variety of *in silico* chemogenomic methods have been developed to predict drug–target or compound–protein interactions on a genome-wide scale (Bleakley and Yamanishi, 2009; Faulon *et al.*, 2008; Jacob and Vert, 2008; Keiser *et al.*, 2009; van Laarhoven *et al.*, 2011; Yamanishi *et al.*, 2008). The underlying idea is that similar ligands are likely to interact with similar proteins, and prediction is performed based on chemical structures of ligand compounds, protein sequences of targets and the currently known compound–protein interactions.

Another promising approach is to use pharmacological information such as drug side effects and adverse drug reactions. The use of side effect similarity has been recently proposed to infer whether two drugs share a target (Campillos *et al.*, 2008). This method requires drug package inserts that describe the detailed side effect information, so it is applicable only to marketed drugs for which side effect information is provided. To overcome this limitation, several methods have been proposed to predict unknown side effects from chemical structures (Atias and Sharan, 2011; Yamanishi *et al.*, 2010). These methods are useful when chemical structures and side effects are correlated with each other to some extent; however, there are still some drug–target interactions that cannot be explained or predicted using these methods.

Recently, the adverse event reporting system (AERS) in the US Food and Drug Administration (FDA) has been gaining a lot of attentions for computational applications of pharmaceutical analyses. AERS is a spontaneous reporting system that routinely collects adverse drug event reports from patients, clinicians and pharmaceutical companies in order to support the FDA's post-marketing safety surveillance program for all approved drugs and biological products. One of the advantages of using AERS over package inserts is that the users need not wait for the sufficient amount of adverse effect incidents to occur in order to be written on the package inserts. An algorithm has been developed to identify hidden drug–drug interactions (DDIs) in adverse event reports (Tatonetti *et al.*, 2012), and the same authors detected DDIs between paroxetine and pravastatin which increases blood glucose levels (Tatonetti *et al.*, 2011). A data-mining technique has been used to analyze adverse event profiles of platinum agents, and it was observed that acute renal failure was also more predominant forcisplatin, and carboplatin did not increase the blood level of creatinine (Sakaeda *et al.*, 2011). These previous works highlight the usefulness of AERS for pharmaceutical and clinical research.

In this article, we propose pharmacological similarity for all possible drugs using AERS and develop a new method to predict unknown drug–target interactions on a large scale from the integration of pharmacological similarity of drugs and genomic sequence similarity of target proteins in the framework of a pharmacogenomic approach. The advantages of using the AERS-based pharmacological similarity stem from the availability of information for a much larger number of drugs, compared with the other more specific resources (e.g. SIDER and JAPIC) used in the previous works, and unexpected drug targets can be identified only through safety surveillance program and adverse drug events reported in the post-marketing study practice. In the results, we predict drug–target interactions involved in many protein families, and show the usefulness of our proposed method for predicting unknown drug–target interactions which cannot be expected from analyzing drug chemical structures. To our knowledge, this is the first report to predict drug targets using AERS. A comprehensive prediction of drug–target interaction networks enables us to suggest new potential drug–target interactions.

## 2 MATERIALS

### 2.1 Pharmacological data

#### 2.1.1 AERS

Side effect keywords (adverse event keywords) for drugs were retrieved from the public release of the adverse event reporting system (AERS) database in the US FDA [http://www.fda.gov/Drugs/GuidanceComplianceRegulatoryInformation/Surveillance/AdverseDrugEffects/default.htm]. We derived 2 904 050 reports containing 291 997 drugs from the period of 2004–2010. Every adverse event report was given an ID (referred to as ISR), and various aspects of the report were stored in several files separately. For example, the file named as REACyyQq.TXT stores the names of side effects in the reports, and DRUGyyQq.TXT stores the drug/biologic information of the medications, where *yy* and *q* mean the year and the quarter, respectively (e.g. *yy* = 10 and *q* = 3 if the file contains the data from the third quarter of 2010). In the file DRUGyyQq.TXT, the suspect drugs for the adverse events were classified into the four categories: primary suspect drug (PS), secondary suspect drug (SS), concomitant (C) and interacting (I). In this study, we used the drugs (compound names or product names) labeled as PS or SS and searched the equivalent drugs registered in the KEGG DRUG database. From the file REACyyQq.TXT, frequency of the keywords were defined as the number of reports (corresponding to the ISR IDs) containing the keyword divided by the number of all reports, and the keywords that appear too frequently (> 0.001) or too rarely (the number of reports were < 5) were removed. The remaining keywords was linked with the KEGG DRUG IDs (also referred to as D numbers). As the result, we obtained the dataset containing 5477 drugs, 11 047 side effect keywords and 1 481 256 associations between them.

We constructed two types of pharmacological profiles for drugs based on the frequency information of side effect keywords in adverse event reports and the binary information (presence or absence) in adverse event reports. We refer to them as AERS-freq and AERS-bit, repsectively, in this study. First, each drug is represented by a real-valued profile **x***_aersfreq_* = (*x*_1_,*x*_2_,···,*x_K_*)^⊤^ where a side effect keyword is coded its frequency in adverse event reports, respectively, across the *K* = 11047 keywords. Second, each drug is represented by a binary profile **x***_aersbit_* = (*x*_1_,*x*_2_,···,*x_K_*)^⊤^ where a side effect keyword is coded 1 or 0, respectively, across the *K* = 11,047 side effect keywords.

#### 2.1.2 SIDER

Side effect keywords for drugs were obtained from the SIDER database [http://sideeffects.embl.de/] which contains information about marketed medicines and their recorded side effects or adverse drug reactions (Kuhn *et al.*, 2010). Each drug is represented by a binary profile **x***_sider_* = (*x*_1_,*x*_2_,···,*x_K_*)^⊤^ in which a side effect keyword is coded 1 or 0, respectively, across about the *K* = 1385 side effect keywords.

#### 2.1.3 JAPIC

Side effect keywords for drugs (pharmaceutical molecules) were obtained from the Japan Pharmaceutical Information Center (JAPIC) database [http://www.japic.or.jp/]. JAPIC manages all package insert information of pharmaceutical products in Japan, under the approval of Health and Welfare Minister of Japan.

We collected 16 853 side effect keywords with XML tags associated with unwanted characteristics of drugs (e.g. side effect, adverse event, caution and warning). Each drug is represented by a binary profile **x***_japic_* = (*x*_1_,*x*_2_,···,*x_K_*)^⊤^ in which a side effect keyword is coded 1 or 0, respectively, across about the *K* = 16853 side effect keywords.

#### 2.1.4 Relationship between different resources

**Fig. 1.**
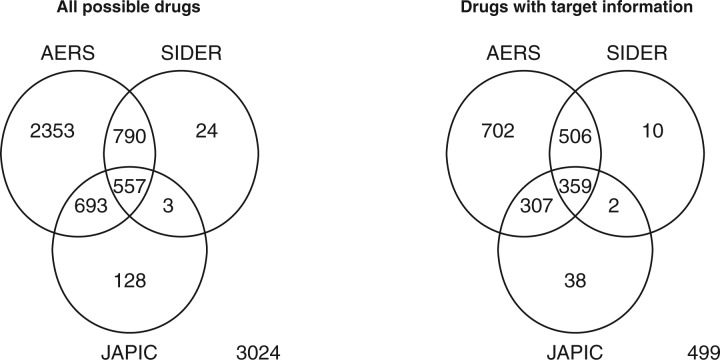
Venn diagram of all possible drugs (left) and drugs with target information (right) across AERS, SIDER and JAPIC.

Drug identifiers are different between AERS, SIDER and JAPIC, which makes it difficult to make a comparison. Therefore, we mapped all possible drugs in the different databases onto the KEGG DRUG database (Kanehisa *et al.*, 2008) based on drug names and product names in order to use unique identifiers for the same drugs. The left panel in Figure 1 shows Venn-diagram of all drugs in KEGG across AERS, SIDER and JAPIC, where 3024 drugs are not assigned any side effect information in AERS, SIDER or JAPIC. The number of drugs with pharmacological information in AERS is much larger than those in SIDER and JAPIC.

### 2.2 Drug–target interaction data

The information about drug–target interactions was obtained from the KEGG DRUG database (Kanehisa *et al.*, 2008). KEGG DRUG includes only the drug–target interactions that are verified well in wet experiments and explained sufficiently in the literature. We did not use drugs whose molecular weights were *<*100 in this study. In our dataset, there are 6769 drug–target interactions consisting of 2423 drugs and 436 target proteins.

The right panel in Figure 1 shows Venn diagram of drugs with target information in KEGG DRUG and with pharmacological information in AERS, SIDER and JAPIC, where 2423 drugs out of 7572 total drugs in KEGG DRUG have target information and the other 5149 drugs do not have any target information. The 2423 drugs, i.e. the all drugs with target information were referred to as ‘all drugs-associated interaction data’ in this study. The number of drugs with target information in AERS is much larger than those in SIDER and JAPIC. There are 359 drugs that are common across AERS, SIDER and JAPIC and have target information in KEGG DRUG, which is referred to as ‘common drugs-associated interaction data’ in this study. The corresponding drug–target interaction data consist of 1188 drug–target interactions, 359 common drugs and 226 target proteins.

### 2.3 Chemical structures and protein sequences

Chemical structures of drugs were obtained from the KEGG DRUG database (Kanehisa *et al.*, 2008). We computed the chemical structure similarities between two drugs **x** and **x**′ using the graph kernel (Mahe *et al.*, 2005). The similarity score is denoted as *s*_chem_(**x**,**x**′) and referred to as ‘chemical similarity’ in this study.

Amino acid sequences of proteins coded in the human genome were obtained from the KEGG GENES database (Kanehisa *et al.*, 2008). We computed the sequence similarities between two proteins **y** and **y**′ using the local alignment kernel (Saigo *et al.*, 2004). The similarity score is denoted as *s*_geno_(**y**,**y**′) and referred to as ‘genomic sequence similarity’ in this study.

## 3 METHODS

### 3.1 Pharmacological similarity

Suppose that each drug is represented by a pharmacological profile **x** = (*x*_1_,*x*_2_,···,*x_K_*)^⊤^, where *K* is the number of side effect keywords. The pharmacological similarity between two drugs **x** and **x**′ is evaluated by the weighted cosine correlation coefficient between the above profiles as
(1)
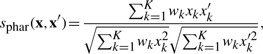

where *w_k_* is the weight function for the *k*th keyword defined as

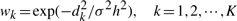

where *d_k_* is the frequency of the *k*th keyword in the data, and *K* is the total number of keywords in the data, *σ* is the mean of {*d_k_*}_*k*=1_^*K*^ and *h* is a parameter (set to 1 in this study). The weight function is introduced to put more emphasis on infrequent keywords rather than frequent keywords, because rare keywords (e.g. agnosia, variant angina, aspergillosis and gouty arthritis) are more informative than common keywords (e.g. nausea, dizziness, vomiting and rash) in terms of phenotypic characteristics of drugs. The similarity score is referred to as ‘pharmacological similarity’ in this study.

The pharmacological similarity functions for drug side effect profiles based on AERS-freq, AERS-bit, SIDER and JAPIC are denoted by *s*_aersfreq_, *s*_aersbit_, *s*_sider_ and *s*_japic_, respectively.

### 3.2 Pairwise kernel regression

Let us now consider the situation where we have a set of drugs 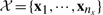, a set of target proteins 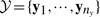, a set of known drug–target interaction pairs *E*^+^ and a set of drug–target pairs whose interactions are unknown *E*^−^, where *n_x_* is the number of drugs and *n_y_* is the number of target proteins in *E*+ ∪ *E*^−^. Given any drug–target pair (**x**,**y**), we want to predict how likely an interaction exists between the drug **x** and the target protein **y**.

We propose a pairwise kernel regression (PKR) model for a pair (**x**,**y**) as
(2)
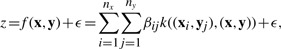

where (**x***_i_*,**y***_j_*) is a drug–target pair in *E*^+^ ∪ *E*^−^, *f* is the projection 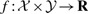, *β_ij_* is a weight element, *k*(·,·) is a kernel similarity function for drug–target pairs and *ε* is a noise term. High-scoring drug–target pairs in *f*(**x**,**y**) are predicted to be interaction pairs.

Let us define *k_x_*(·,·) as a kernel similarity function for drugs and *k_y_*(·,·) as a kernel similarity function for target proteins. Using the following property of the tensor product kernel, the pairwise kernel *k* can be represented by the product of kernel *k_x_* and kernel *k_y_* as follows:

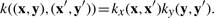

Then, the PKR model can be represented by
(3)
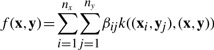

(4)
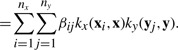

This operation enables us to avoid working on the pairwise space, otherwise computational cost in the pairwise space is prohibitive.

For the training set of drug–target pairs (**x***_i_*,**y***_j_*) (*i* = 1,···,*n_x_*,*j* = 1,···,*n_y_*), we propose to set *z_ij_* as follows:
(5)
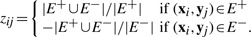

For the test set of drug–target pairs (to be predicted), we set *z_ij_* to 0 in the initial stage of the learning process. The fitting of the model can be done by finding *β_ij_* that minimizes the following loss function:
(6)
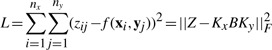

(7)
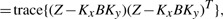

where *K_x_* is an *n_x_* × *n_x_* kernel similarity matrix (*K_x_*)*_ij_* = *k_x_*(**x***_i_*,**x***_j_*), *K_y_* is an *n_y_* × *n_y_* kernel similarity matrix (*K_y_*)*_ij_* = *k_y_*(**y***_i_*,**y***_j_*) and *Z* and *B* are matrices defined as (*Z*)*_ij_* = *z_ij_* and (*B*)*_ij_* = *β_ij_*(*i* = 1,2,···,*n_x_*,*j* = 1, 2,···, *n_y_*).

Taking the differential of *L* with respect to *B* and setting it to zero, the weight matrix *B* can be analytically obtained by solving
(8)
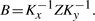

Once we have trained our model, which is computing *B*, we can apply the model to unseen object pairs (drug–target pairs in this case). We can introduce a regularization by applying the singular value decomposition to *Z* as *Z* ≈ *U_q_D_q_V_q_^T^*, where *D_q_* is a diagonal matrix of *q* singular values, and *U_q_* and *V_q_* are matrices of singular vectors associated with the *q* singular values. In this case, the weight matrix *B* can be obtained by solving *B* = *K_x_*^−1^ *U_q_D_q_V_q_^T^ K_y_*^−1^.

### 3.3 Predictive models for drug–target interactions

We consider five approaches as follows:

- AERS-freq-based pharmacogenomic approach (AERS-freq): The prediction is performed based on AERS-freq-based pharmacological similarity *s*_aersfreq_ and sequence similarity *s*_geno_.
- AERS-bit-based pharmacogenomic approach (AERS-bit): The prediction is performed based on AERS-bit-based pharmacological similarity *s*_aersbit_ and sequence similarity *s*_geno_.
- SIDER-based pharmacogenomic approach (SIDER): The prediction is performed based on SIDER-based pharmacological similarity *s*_sider_ and sequence similarity *s*_geno_.
- JAPIC-based pharmacogenomic approach (JAPIC): The prediction is performed based on JAPIC-based pharmacological similarity *s*_japic_ and sequence similarity *s*_geno_.
- Chemogenomic approach (CHEM): The prediction is performed based on chemical similarity *s*_chem_ and sequence similarity *s*_geno_.

To investigate the effect of data integration, we also consider two integrative approaches as follows:

- Integrated pharmacogenomic approach (INTEG-P): The prediction is performed based on the integrated pharmacological similarity *s*_aersfreq_ + *s*_sider_ + *s*_japic_ and sequence similarity *s*_geno_.
- Integrated pharmaco-chemogenomic approach (INTEG-PC): The prediction is performed based on the integrated similarity *s*_aersfreq_ + *s*_sider_ + *s*_japic_ + *s*_chem_ and sequence similarity *s*_geno_.

### 3.4 Experimental protocol

There are mainly two practical situations for drug target identification. The first situation is that the drug has at least one known target protein and we want to detect unknown additional target proteins (e.g. off-targets) of the drug. The second situation is that the drug has no known target and we want to predict all the potential target proteins of the drug. From the viewpoint of the above two practical situations, we perform two types of cross-validations: pair-wise cross-validation and block-wise cross-validation.

In the pair-wise cross-validation we performed the following 3-fold cross-validation. (i) We randomly split drug–target pairs in the gold standard data into three subsets of roughly equal sizes by pair. (ii) We took each subset as a test set and the remaining two subsets as a training set. (iii) We trained a predictive model on the training set. (iv) We computed the prediction scores for drug–target pairs in the test set. (v) Finally, we evaluated the prediction accuracy over the 3-folds.

In the block-wise cross-validation we performed the following 3-fold cross-validation. (i) We randomly split drugs in the gold standard data into three drug subsets. (ii) We took each drug subset, and constructed a test set of drug–target pairs from the drug subset and all targets and a training set of drug–target pairs from the remaining two drug subsets and all targets. (iii) We trained a predictive model on the training set. (iv) We computed the prediction scores for drug-target pairs in the test set. (v) Finally, we evaluated the prediction accuracy over the 3-folds. Note that only drugs are split into a training set and a test set, and targets are common across training set and test set.

There are many drugs that were optimized from the same drug lead, so these drugs are chemically and structurally similar to each other. If these drugs were split into a training set and a test set in the cross-validation, the target prediction would be very easy, which would overestimate the prediction accuracy. To avoid such a trivial prediction, we proposed a clustering of drugs based on their chemical structures and used only representative drugs which are chemically diverse. First, we performed a hierarchical clustering of all drugs in the gold standard data based on their chemical structure similarity scores using single linkage algorithm. Second, we grouped drugs whose chemical structure similarity is greater than a threshold into the same cluster, and we randomly selected only one drug from each cluster. Third, we constructed a set of representative drugs whose chemical structure similarities were less than a threshold. Finally, we prepared several benchmark datasets consisting of only representative drugs by varying the threshold from 0.1 to 1 by 0.1 increments on the hierarchical clustering tree and used them to examine the effect of the threshold on prediction performance.

## 4 RESULTS

### 4.1 Performance evaluation

We tested seven methods: AERS-freq, AERS-bit, SIDER, JAPIC, CHEM, INTEG-P and INTEG-PC on their abilities to predict drug–target interactions by performing two types of 3-fold cross-validations (see Section 3.4 for more details). We used two gold standard datasets: ‘common drugs-associated interaction data’ and ‘all drugs-associated interaction data’ (see the Section 2). For drugs with no available data in each method, we set the missing elements in the corresponding drug kernel similarity matrix to zero.

We evaluated the method performance via the receiver operating characteristic (ROC) curve, which is a plot of true positives as a function of false positives based on various thresholds, where true positives are correctly predicted interactions and false positives are predicted interactions that are not present in the gold standard interactions. We summarized the performance by an area under the ROC curve (AUC) score, where 1 is for a perfect inference and 0.5 is for a random inference. The parameters involved in each method were optimized with the AUC score as the objective function. To obtain a robust result, we repeated the cross-validation experiment five times and computed the average and standard deviation (SD) of the AUC scores over the five repetitions.

Table 1 shows the AUC scores by the pair-wise cross-validation, where the SD values are not shown in the table for easier readability, because most SD values are *<* 0.01. As shown in the upper half of Table 1, the pharmacogenomic approach worked better than the chemogenomic approach for the common drug-associated interaction data. Among the four pharmacological similarities, AERS-bit worked the best in the case of low chemical similarity thresholds, and JAPIC worked the best in the case of high chemical similarity thresholds. It seems that there is little difference between the performance of AERS-freq and AERS-bit. On the other hand, as shown in the bottom half of Table 1 for the all drugs-associated interaction data, the pharmacogenomic approach worked better than the chemogenomic approach in the case of low chemical similarity thresholds, but worse than the chemogenomic approach in the case of high chemical similarity thresholds. One explanation is that chemical structure information is available for most drugs, so the number of predictable drugs by the chemogenomic approach is larger than that of the pharmacogenomic approach, which suggests an advantage of the chemogenomic approach over the pharmacogenomic approach in terms of prediction coverage of drug–target interactions. In addition, there are many drug derivatives from the same drug lead that are chemically and structurally similar with each other within the all drug-associated interaction data, so the prediction with chemical structures are relatively easy in such a case.

**Table 1.** AUC scores in the 3-fold pair-wise cross-validation

Data type	Clustering threshold (number of representative drugs)	AERS-freq	AERS-bit	SIDER	JAPIC	CHEM	INTEG-P	INTEG-PC
Common drugs	0.1 (3)	0.836	**0.838**	0.824	0.835	0.833	0.832	0.832
Common drugs	0.2 (15)	0.947	0.953	0.949	**0.964**	0.946	0.957	0.956
Common drugs	0.3 (32)	0.962	0.959	0.957	**0.965**	0.961	0.962	0.963
Common drugs	0.4 (68)	0.941	0.935	0.931	**0.953**	0.925	0.947	0.947
Common drugs	0.5 (103)	0.953	0.949	0.944	**0.961**	0.943	0.958	0.955
Common drugs	0.6 (143)	0.963	0.960	0.956	**0.976**	0.954	0.972	0.970
Common drugs	0.7 (178)	0.966	0.964	0.944	**0.980**	0.959	0.976	0.975
Common drugs	0.8 (214)	0.956	0.958	0.956	**0.981**	0.958	0.976	0.976
Common drugs	0.9 (249)	0.955	0.954	0.954	**0.978**	0.960	0.975	0.973
Common drugs	1.0 (359)	0.971	0.971	0.964	**0.984**	0.972	0.983	0.982
All drugs	0.1 (90)	0.840	**0.844**	0.749	0.834	0.751	0.856	0.866
All drugs	0.2 (110)	**0.879**	**0.879**	0.828	0.862	0.832	0.882	0.885
All drugs	0.3 (157)	0.930	0.934	0.932	**0.942**	0.935	0.941	0.946
All drugs	0.4 (272)	**0.924**	0.923	0.904	0.923	0.920	0.930	0.935
All drugs	0.5 (434)	0.928	0.929	0.919	**0.934**	0.926	0.939	0.942
All drugs	0.6 (621)	0.922	0.922	0.921	**0.932**	0.930	0.935	0.940
All drugs	0.7 (786)	0.930	0.932	0.922	**0.938**	0.936	0.941	0.950
All drugs	0.8 (948)	0.928	0.928	0.924	0.935	**0.944**	0.942	0.952
All drugs	0.9 (1444)	0.927	0.930	0.924	0.938	**0.948**	0.945	0.958
All drugs	1.0 (2368)	0.930	0.931	0.923	0.920	**0.962**	0.942	0.962

Bold indicates the best result between AERS-freq, AERS-bit, SIDER, JAPIC and CHEM in each clustering threshold.

Table 2 shows the AUC scores by the block-wise cross-validation, where ‘NA’ means that the algorithm did not work because it detected almost no similarities between drugs in the training set. Similar tendencies exhibited in the pair-wise cross-validation can be observed in the block-wise cross-validation as well. However, the AUC scores in the block-wise cross-validation tend to be much lower than those in the pair-wise cross-validation. This result suggests that predicting all potential targets of drugs which have no known targets is much more difficult than predicting missing targets (e.g. off-targets) of drugs with known targets (which have at least one known target protein). Among the four pharmacological similarities for the common drug-associated interaction data, SIDER worked the best in the case of low chemical similarity thresholds, and JAPIC worked the best in the case of high chemical similarity thresholds. Among the four pharmacological similarities for the all drugs-associated interaction data, AERS-freq or AERS-bit work the best, because the number of drugs with AERS-based pharmacological information is much larger than those of SIDER-based or JAPIC-based pharmacological information. These results suggest that AERS-freq-based pharmacological similarity is useful for predicting unknown drug–target interactions which are not expected from drug chemical structures.

**Table 2.** AUC scores in the 3-fold block-wise cross-validation

Data type	Clustering threshold (number of representative drugs)	AERS-freq	AERS-bit	SIDER	JAPIC	CHEM	INTEG-P	INTEG-PC
Common drugs	0.1 (3)	0.663	0.701	**0.776**	0.705	0.580	0.780	0.749
Common drugs	0.2 (15)	0.636	0.721	0.613	**0.740**	0.702	0.650	0.675
Common drugs	0.3 (32)	0.698	0.680	0.714	**0.733**	0.662	0.711	0.708
Common drugs	0.4 (68)	0.716	0.704	0.712	**0.810**	0.610	0.774	0.770
Common drugs	0.5 (103)	0.678	0.644	0.680	**0.788**	0.684	0.766	0.741
Common drugs	0.6 (143)	0.773	0.751	0.778	**0.848**	0.671	0.841	0.829
Common drugs	0.7 (178)	0.789	0.758	0.740	**0.865**	0.724	0.840	0.818
Common drugs	0.8 (214)	0.805	0.786	0.816	**0.912**	0.794	0.891	0.882
Common drugs	0.9 (249)	0.820	0.792	0.807	**0.910**	0.813	0.899	0.897
Common drugs	1.0 (359)	0.862	0.852	0.851	**0.943**	0.869	0.934	0.931
All drugs	0.1 (90)	**0.820**	0.819	NA	0.713	0.523	0.846	0.854
All drugs	0.2 (110)	**0.720**	0.717	0.470	0.640	0.464	0.729	0.696
All drugs	0.3 (157)	**0.720**	0.703	0.516	0.647	0.658	0.711	0.747
All drugs	0.4 (272)	0.733	0.732	0.552	0.684	**0.749**	0.748	0.812
All drugs	0.5 (434)	0.736	0.742	0.588	0.706	**0.783**	0.760	0.831
All drugs	0.6 (621)	0.767	0.768	0.646	0.750	**0.826**	0.823	0.874
All drugs	0.7 (786)	0.766	0.765	0.651	0.750	**0.834**	0.826	0.883
All drugs	0.8 (948)	0.768	0.765	0.647	0.749	**0.840**	0.825	0.879
All drugs	0.9 (1144)	0.774	0.760	0.661	0.748	**0.859**	0.833	0.902
All drugs	1.0 (2368)	0.841	0.849	0.724	0.723	**0.939**	0.885	0.950

Bold indicates the best result between AERS-freq, AERS-bit, SIDER, JAPIC and CHEM in each clustering threshold.

We did not observe any positive effect of data integration in the cross-validation for the common drug-associated interaction data, but observed some positive effects in the cross-validation for the all drug-associated interaction data. Predictable drugs in each method depend on the coverage of drugs in each database in the all drug-associated interaction data, so different data sources might complement each other. INTEG-P worked better than individual pharmacogenomic approaches in most cases, which suggests the usefulness of integrating different side-effect resources. INTEG-PC worked the best in most cases, but it was at a competitive level with AERS-freq or AERS-bit in the case of low clustering thresholds. This result suggests that the integration of pharmacological information and chemical information is useful only in the prediction of potential targets of drugs sharing chemical similarity to some extent with drugs in the training set.

We also conducted the same cross-validation experiments using the precision–recall curve (PR curve) and the area under the PR curve (AUPR). The resulting AUPR scores are shown in Supplemental Table 1, and the same tendencies exhibited in the AUC scores can be observed in the AUPR scores as well.

### 4.2 Prediction for unknown drug–target interactions

Having confirmed the usefulness of our method, we conducted a comprehensive prediction of interactions between all possible drugs and target proteins. We trained a predictive model using all known drug–target interactions in the gold standard data and predicted unknown targets for all possible drugs for which pharmacological information was available in AERS. We predicted potential off-targets of 1874 drugs with known targets in the gold standard data and potential target profiles of 2519 uncharacterized drugs in KEGG DRUG (which were absent from the gold standard data mainly because target proteins have not yet been identified) using the AERS-freq-based pharmacological similarity. All the prediction results can be obtained from the supplemental materials.

**Fig. 2.**
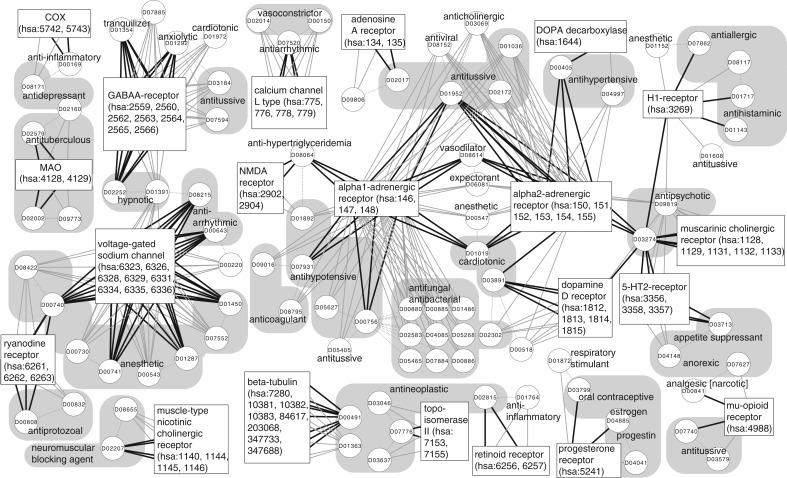
Part of drug–target interaction network obtained from AERS-freq in this study. Circles and rectangles indicate drugs and target proteins, respectively, where the drugs are represented by the KEGG DRUG IDs. Black bold lines indicate known drug–target interactions. Gray solid lines and dotted lines indicate predicted (score ≥ 100) drug–target interactions and the similar drug pairs, respectively. Drugs with similar efficacy were located as close as possible and shadowed where possible

We focused on drug–target interactions that were predicted only by the AERS-freq-based pharmacogenomic approach (which were not predicted by the previous chemogenomic or other pharmacogenomic approaches). Figure 2 shows some examples of known and predicted (score ≥ 100) drug–target interaction networks obtained by the AERS-freq-based method. Although the target prediction was made mainly based on the side effect similarity, drugs with similar efficacy were generally predicted to target the same proteins, such as antituberculous agent D09773 (isoniazid glucuronate), hypnotic agent D01391 (bromovalerylurea), anesthetic agents D08422 (procaine), D00730 (butamben), D00543 (enflurane), D07552 (bupivacaine), antiprotozoal agent D00832 (melarsoprol), neuromuscular blocking agent D08655 (tubocurarine), antihypotensive drug D05627 (propatylnitrate), antihypertensive drug D04997 (methyldopate), antineoplastic agents D01363 (carboplatin), D03046 (fosteabine), D03637 (cytarabine), antihistaminic drug D08117 (levocabastine), antipsychotic drug D09819 (chlorpromazine tannate), antitussive drug D03579 (codeine polistirex) and many other drugs that are not shown in Figure 2.

On the other hand, some drugs with different efficacy were predicted to share the same targets, because of similar side effects. Many of them were predicted reasonably, e.g. antitussive agent D01608 (clobutinol) predicted to interact with H1-receptor (hsa:3269) based on the known antihistaminic drug D01143 (isothipendyl). Some other drugs were predicted to interact with the proteins that work in related functions. For example, vasoconstrictor drugs D00150 (angiotensin II) and D02014 (human type angiotensin II) were predicted to interact with L type calcium channel proteins (hsa:775, 776, 778, 779) that are the targets of antiarrhythmic drug D07520 (bepridil). Although it may not be possible for D00150 and D02014 to bind with L type calcium channel directly, this result may indicate the indirect relationships between the vasoconstrictor drugs and the antiarrhythmic drug. Some results may just show synergistic effects, such as those in connections antihypertensive/alpha2-adrenergic receptor/anticholinergic (Fig. 2).

One of the interesting interactions predicted in this study was between the anti-obesity agent D07627 (cathine) and mu-opioid receptor (hsa:4988). As the mu refers to morphine, mu-opioid receptor has high affinity with morphine and many other narcotic drugs such as D07740 (codeine). D07627 was predicted to interact with mu-opioid receptor based on the side-effect similarity with narcotic analgesic drug D00841 (levorphanol). In fact, D07627 is known as a psychoactive stimulant, which is listed in the World Anti-Doping Agency's list of prohibited substances used for the Olympic games. We cannot prove these predicted interactions are valid without wet experiments; however, our manual investigation demonstrated that the proposed method provided convincing predictions.

## 5 DISCUSSION AND CONCLUSION

To our knowledge, this is the first report to predict drug-target interactions using AERS. In this article, we demonstrated the usefulness of our proposed method to predict drug–target interactions that could not be expected from drug chemical structures. This indicates possible advantages of our method when dealing with pharmaceuticals whose chemical structures are not available, such as peptide drugs and crude drug extracts whose medically effective ingredients are not still clear.

Drug–target interactions have been investigated by a variety of statistical or machine learning methods in the context of chemogenomics. Most algorithms in previous chemogenomic methods can be made applicable to this study by replacing the chemical similarity by the pharmacological similarity. Thus we tested the previous algorithms: BLM (Bleakley and Yamanishi, 2009), P-SVM (Faulon *et al.*, 2008; Jacob and Vert, 2008), KRM (Yamanishi *et al.*, 2008) and GIP (van Laarhoven *et al.*, 2011) for the pharmacogenomic problem addressed in this study (AERS-based pharmacological similarity, protein sequence similarity and drug–target interaction data for the common drugs were used), and made the performance comparisons with the proposed PKR algorithm. Supplementary Table S2 shows the resulting AUC score and computational cost for each algorithm, suggesting that the proposed algorithm outperforms the other algorithms in terms of prediction accuracy and computational efficiency. Therefore we used PKR in this study, but there would be little significant difference in the tendencies observed in the results if we used different algorithms.

We showed the value of the AERS data for large-scale prediction of drug–target interaction networks. The future improvement of the prediction method would be provided by more sophisticated design of similarity functions and well-designed text-mining techniques. For examples, protein similarity based on ligand-binding sites and drug side effect similarity based on the tf-idf measures would be interesting. Needless to say, continuous management and further development of the drug databases as well as the collaborative sharing of knowledge may contribute to better prediction of drug–target interaction networks, potentially solving many other pharmaceutical problems.

*Funding:* In part by the Education Unit for Global Leaders in Advanced Engineering and Pharmaceutical Sciences (GL Education Unit) at Kyoto University, JSPS International Training Program (ITP) and the Japan Science and Technology Agency. Computational resources were provided by Bioinformatics Center, Institute for Chemical Research, Kyoto University.

## References

[B1] Atias N., Sharan R. (2011). An algorithmic framework for predicting side-effects of drugs. J. Comput. Biol..

[B2] Blagg J. (2006). Structure-activity relationships for *in vitro* and *in vivo* toxicity. Annu. Rep. Med. Chem..

[B3] Bleakley K., Yamanishi Y. (2009). Supervised prediction of drug-target interactions using bipartite local models. Bioinformatics.

[B4] Campillos M. (2008). Drug target identification using side-effect similarity. Science.

[B5] Dobson C. (2004). Chemical space and biology. Nature.

[B6] Faulon J. (2008). Genome scale enzyme-metabolite and drug–target interaction predictions using the signature molecular descriptor. Bioinformatics.

[B7] Jacob L., Vert J.-P. (2008). Protein–ligand interaction prediction: an improved chemogenomics approach. Bioinformatics.

[B8] Kanehisa M. (2008). KEGG for linking genomes to life and the environment. Nucleic Acids Res..

[B9] Kanehisa M. (2006). From genomics to chemical genomics: new developments in KEGG. Nucleic Acids Res..

[B10] Keiser M. (2009). Predicting new molecular targets for known drugs. Nature.

[B11] Kuhn M. (2010). A side effect resource to capture phenotypic effects of drugs. Mol. Syst. Biol..

[B12] Mahe P. (2005). Graph kernels for molecular structure-activity relationship analysis with support vector machines. J. Chem. Inf. Model..

[B13] Saigo H. (2004). Protein homology detection using string alignment kernels. Bioinformatics.

[B14] Sakaeda T. (2011). Adverse event profiles of platinum agents: data mining of the public version of the FDA adverse event reporting system, AERS, and reproducibility of clinical observations. Int. J. Med. Sci..

[B15] Stockwell B. (2000). Chemical genetics: ligand-based discovery of gene function. Nat. Rev. Genet..

[B16] Tatonetti N. (2011). Detecting drug interactions from adverse-event reports: interaction between paroxetine and pravastatin increases blood glucose levels. Clin. Pharmacol. Ther..

[B17] Tatonetti N. (2012). A novel signal detection algorithm for identifying hidden drug–drug interactions in adverse event reports. J. Am. Med. Inform. Assoc..

[B18] van Laarhoven T. (2011). Gaussian interaction profile kernels for predicting drug–target interaction. Bioinformatics.

[B19] Whitebread S. (2005). Keynote review: *in vitro* safety pharmacology profiling: an essential tool for successful drug development. Drug Discov. Today.

[B20] Yamanishi Y. (2008). Prediction of drug–target interaction networks from the integration of chemical and genomic spaces. Bioinformatics.

[B21] Yamanishi Y. (2010). Drug–target interaction prediction from chemical, genomic and pharmacological data in an integrated framework. Bioinformatics.

